# Exploring the FTIR, Optical and Nuclear Radiation Shielding Properties of Samarium-Borate Glass: A Characterization through Experimental and Simulation Methods

**DOI:** 10.3390/nano11071713

**Published:** 2021-06-29

**Authors:** Shams A. M. Issa, Hesham M. H. Zakaly, Huseyin O. Tekin, Heba A. Saudi, Ali Badawi, Mariia Pyshkina, Gulfem Susoy, Ahmed I. Elazaka, Antoaneta Ene

**Affiliations:** 1 Physics Department, Faculty of Science, Al-Azhar University, Assiut 71524, Egypt; sh_issa@ut.edu.sa; 2 Physics Department, Faculty of Science, University of Tabuk, Tabuk 47512, Saudi Arabia; 3 Institute of Physics and Technology, Ural Federal University, 620002 Yekaterinburg, Russia; maria1pyshkina@gmail.com; 4 Medical Diagnostic Imaging Department, College of Health Sciences, University of Sharjah, Sharjah 27272, United Arab Emirates; htekin@sharjah.ac.ae; 5 Medical Radiation Research Center (USMERA), Uskudar University, 34672 Istanbul, Turkey; 6 Department of Physics, Faculty of Science, Al-Azhar University (Girls’ Branch), Nasr City 11884, Egypt; heba_saudi@azhar.edu.eg; 7 Department of Physics, College of Science, Taif University, P.O. Box 11099, Taif 21944, Saudi Arabia; daraghmeh@tu.edu.sa; 8 Department of Physics, University College of Turabah, Taif University, P.O. Box 11099, Taif 21944, Saudi Arabia; 9 Department of Physics, Faculty of Science, Istanbul University, 34452 Istanbul, Turkey; glfmsusoy972@gmail.com; 10 Institute of Nuclear Physics and Engineering, National Research Nuclear University MEPhI, 115409 Moscow, Russia; ahmed_ismailel@azhar.edu.eg; 11 Physics Department, Faculty of Science, Al-Azhar University, Cairo 11884, Egypt; 12 INPOLDE Research Center, Department of Chemistry, Physics and Environment, Faculty of Sciences and Environment, Dunarea de Jos University of Galati, 47 Domneasca Street, 800008 Galati, Romania

**Keywords:** Sm-B_2_O_3_ glass, optical property, radiation attenuation property, MC simulation

## Abstract

(Tl_2_O_3_)_30_-(Li_2_O)_10_-(B_2_O_3_)_(60−*y*__)_-(Sm_2_O_3_)*_y_* glass system with various Sm_2_O_3_ additives (*y* = 0, 0.2, 0.4, 0.6) was studied in detail. The vibrational modes of the (Tl_2_O_3_)_30_-(Li_2_O)_10_-(B_2_O_3_)_(60−*y*)_ network were active at three composition-related IR spectral peaks that differed from those mixed with Samarium (III) oxide at high wavenumber ranges. These glass samples show that their permeability increased with the Samarium (III) oxide content increase. Additionally, the electronic transition between localized states was observed in the samples. The MAC, HVL, and Zeff values for radiation shielding parameters were calculated in the energy range of 0.015–15 MeV using the FLUKA algorithm. In addition, EBF, EABF, and Σ_R_ values were also determined for the prepared glasses. These values indicated that the parameters for shielding (MAC, HVL, Z_eff_, EBF, EABF, and Σ_R_) are dependent upon the Samarium (III) oxide content. Furthermore, the addition of Samarium (III) oxide to the examined glass samples greatly reinforced their shielding capacity against gamma photon. The findings of the current study were compared to analyses of the XCOM software, some concretes, and lead. In the experiment, it was found that the SMG0.6 glass sample was the strongest shield.

## 1. Introduction

Radioactivity is created by naturally occurring radioactive sources. These radioactive sources are found in the Earth’s crust (soil, air, water, plants, and other creatures). Radioactivity is emitted by human activity and is used in various fields, such as medicine, industry, agriculture, livestock, daily-use products, research, and nuclear power [1]. Since radiation was first identified as harmful to humans, scientists began to take precautions against it, especially penetration by higher intensity gamma radiation. A radiation dosage should be kept as low as possible; for this purpose, dense shielding fabrics have been used for hazardous waste, industrial waste, and biological waste. The most frequently used metal is lead because of its density and high atomic number. There are various types of lead material, such as dust and glass [2]. Pure Pb is smooth and hard, but its major disadvantages include toxicity, weight, and secondary ionizing radiation. Since lead is not biologically inert, its toxic effect cannot be eliminated. The evolution of high-performance radiation facilities is critical for radiation protection, especially for consolidated systems and mobile equipment such as naval nuclear propulsion systems, wagon-mounted neutron sources, space-based nuclear power reactors, and other sophisticated nuclear systems [3,4]. The main purpose of using a radiation shield is to reduce radiation emission and, for this reason, a shield must be put around nuclear facilities [5]. Since space in nuclear facilities is limited, the shield must be reinforced, light, and specific [6]. Major factors in the physical features of materials are their chemical composition, bonding forces, and density [7]. As concrete has various benefits (ease of fabrication and low cost), it is widely used for radiation safety. Unfortunately, these materials are prone to cracking over time due to the stresses generated by stretching. Moreover, these cracks are invisible [8]. Glass materials have been suggested as an alternative material for protection from radiation because they resist the defects in lead materials. Glass materials stand out in many applications due to their high transparency, high chemical resistance, and good radiation-protection properties. Because of their super absorption of gamma radiation, glass materials that contain lead (Pb) are commonly used in radiation safety applications [9]. However, as Pb is environmentally and biologically harmful, glass products without Pb must be used as a substitute [10]. Glass-forming oxides like borates are commonly used in technological applications. Borate is one of the top mineral glass materials, as it helps glass solidify and improve glass quality through improved clarity, refractive index, and rare earth ion solubility and hardness. Samarium (Sm), a rare-earth ion, can be used as an additive in various crystal hosts and can also be utilized as a glass host for intense emissions in the visible region. Samarium has a high neutron absorption capacity. On the other hand, Li_2_O-Lithium oxide, one of the oxides used in glassmaking, is a plasticizer. Li_2_O is especially preferred in the production of special glasses and is widely used as a regulator in glass production. In addition, Tl_2_O_3_ has photocatalytic and thermochromic properties. It absorbs visible light, has a moderate electrical conductivity, and can function as a transparent conductor [11]. Therefore, the Sm_2_O_3_-B_2_O_3_-Tl_2_O_3_-Li_2_O glass sample has been investigated as a potentially promising glass system in gamma ray and neutron radiation shielding. All technical details of the study and its experimental and theoretical basis are explained later in this article. It should be emphasized that the findings of this study may be applicable to future uses of the glass types tested and investigated for nuclear radiation protection. In addition, as can be clearly seen from the obtained results, the positive effect of Samarium (III) oxide addition on the nuclear shielding performances of the examined SMG0.0, SMG0.2, SMG0.4, and SMG0.6 glass samples are understood more clearly.

## 2. Methods and Materials

**Figure 1:**
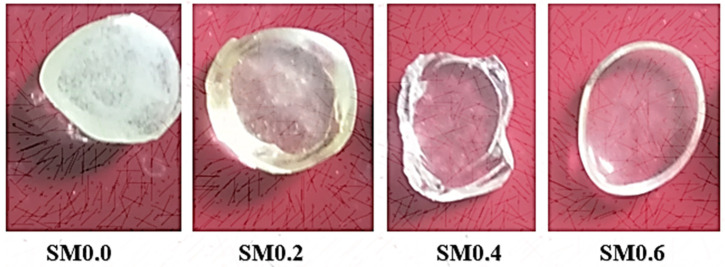
Fabricated (Tl_2_O_3_)_30_-(Li_2_O)_10_-(B_2_O_3_)_(60−*y*)_-(Sm_2_O_3_)*_y_* glass system.

Glass samples in the (Tl_2_O_3_)_30_-(Li_2_O)_10_-(B_2_O_3_)_(60−*y*)_-(Sm_2_O_3_)*_y_* system, where *y* values are given as 0, 0.2, 0.4, and 0.6 wt.%, were equipped by use of melt quenching technique at 1200 °C in an electric furnace in a porcelain pot. After this, the dry oxygen was bubbled for 2 h. Then, these mixtures were poured into stainless steel molds and allowed to cool to form flat, round shapes. After the quenching process was completed, the glasses had to be cooled to room temperature. Initially, annealing was carried out at a temperature under the transition temperature of the proposed glass system for 3 h at 300 °C to reduce thermal stress (Figure 1).

**Figure 2:**
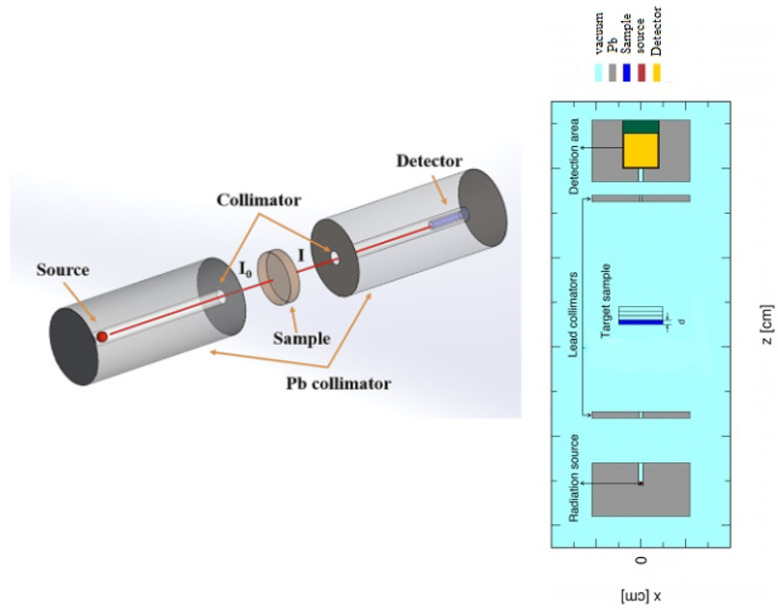
Simulation FLUKA setup for incident gamma radiation with glass samples.

The Archimedes principle was used to determine the molar volume and density of the processed samples. The weight measurements of these samples were produced in toluene and air (at room temperature ρ_liq_ = 0.866 g/cm^3^) with a 4-digit precision microbalance. At room temperature, the absorbance (A) value was determined using a Shimadzu UV-2101 spectrometer (Kyoto, Japan) over a wavelength range of 190–1100 nm. FLUKA is one of the Monte Carlo radiation transport programs [12]. It is a multipurpose Monte Carlo program developed to calculate particle transport and the interaction of particles with matter. FLUKA has the ability to simulate electromagnetic and hadronic interactions and particle transport in any target material. Each command contains one or more lines [13]. This line is also called the card. Each geometric region is covered with a homogeneous material or vacuum; the materials can be simple elements or compounds. In the entry file, the materials are identified with the MATERIAL entry card. Here, the atomic number of the materials, their atomic mass, density, and the name of the material are given. If the material used is a compound, the COMPOUND card must be attached to the MATERIAL card. The density, name, and number of the compound are then written to the entry file. The purpose of FLUKA and similar Monte Carlo simulation codes is to analyze the interactions of charged particles or radiation with matter. The interactions of primary particles, entering the system by passing through various materials, are calculated individually during the simulation and obtained depending on the ‘detector’ cards specified in the user’s input file. A cylindrical-shaped NaI scintillation detector (3 inches × 3 inches) was put in a Pb-cylindrical collimator with an outer diameter of 12 cm, an interior diameter of 0.2 cm and a length of 13 cm. There are many types of detectors in FLUKA; USRBDX, USRBIN, USRTRACK, USRYIELD are the most widely used detector types. A USRTRACK scorecard defined the NaI area as the track length variation. USRTRACK is used to obtain energy spectra depending on each track length selected during the tracking of particles within a region. It is also possible to determine the average differential flux of particles within the region. According to the statistical error (<1%), the total number of primary photons simulated ranged between 10 and 20 million. The scattering of a photon inside the volume of the detector was determined using a USRBIN card. USRBIN is used to calculate the energy stored in a particular region, known as integrated fluxes. It provides results with three-dimensional, full-color images. A BEAM card was then used to characterize the particle shape and energy. A BEAM card was created to determine the beam source in this investigation to establish a monoenergetic photon (0.2 cm in diameter) at energy values of 0.081, 0.356, 0.662, 1.173, and 1.33 MeV. Additionally, a BEAMPOS card was created to adjust the beam source direction and location in the plus z-axis. The coordinates of the beam spot center and the beam direction are defined on the BEAMPOS card [14,15,16]. At low energy levels, the photon transmission power reduction was regulated to a value of 10^−7^ GeV using an EMFCUT card. EMFCUT contributes to the determination of both the energy thresholds for electron and photon creation in various materials and the interruptions in electron and photon transit in specified locations. The cylindrical shape, with a diameter of 4 cm and thicknesses ranging over 0.02–0.2 cm, was employed to simulate glass samples. The samples were sorted based on RPP body shape. The rectangular parallelepiped-RPP is illustrated by six numbers called X-axis, Y-axis, and Z-axis, and defined by the perpendicular sides. Best results were achieved using +5 cm and −5 cm values for Xmax (Ymax) and Xmin (Ymin) [17]. As a consequence, a target material 15 cm in length and width was developed in varying thicknesses, and it was named Zmax and Zmin. The detector chamber was then filled with photons that interacted with the sample. A Pb collimator was added to obscure the scattering photons. However, the FLUKA algorithm was used to forecast MAC values by obtaining both the number of photons traveling through the material and the starting photon count in the detector volume. Additionally, USRBIN was used as a detector card, allowing the estimation of photon flux inside detector volume (Figure 2).

## 3. Results and Discussion

### 3.1. Investigations on Structural Properties

**Figure 3:**
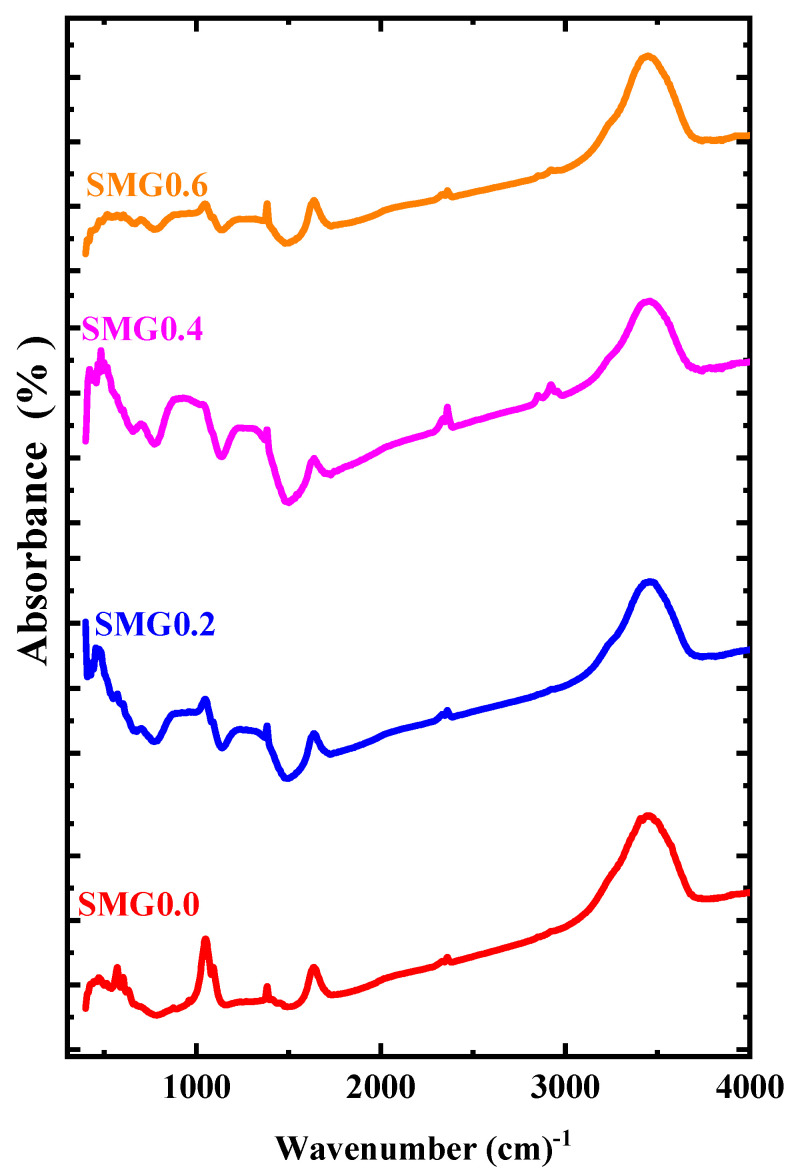
FTIR spectra of glass samples.

The vibration-manners of the 60B_2_O_3_-30Tl_2_O_3_-10Li_2_O glass system are effective at three different IR spectral peaks in the high range of wavenumber (Figure 3).

These values are 1035-1643-3434 cm^−1^, respectively. The wavenumber range between 850–1200 cm^−1^ is related to the B–O stretching of tetrahedral ${\mathrm{BO}}_{4}^{-}$ units [18,19,20]. The peak absorption of 1643 cm^−1^ is related to the H–O–H bending mode and crystal water [21]. On the other hand, wide composite bands near the infrared region (3200–3800 cm^−1^) originate from the hydroxyl or B-OH groups found in the samples [22,23]. A new peak representing Sm-O bonds appeared in the glass system (Tl_2_O_3_)_30_-(Li_2_O)_10_-(B_2_O_3_)_(60−y)_-(Sm_2_O_3_)*_y_* (*y* = 0.2, 0.4, 0.6) at 2355 cm^−1^, as shown in Table 1.

### 3.2. Investigations on Optical Properties

**Figure 4:**
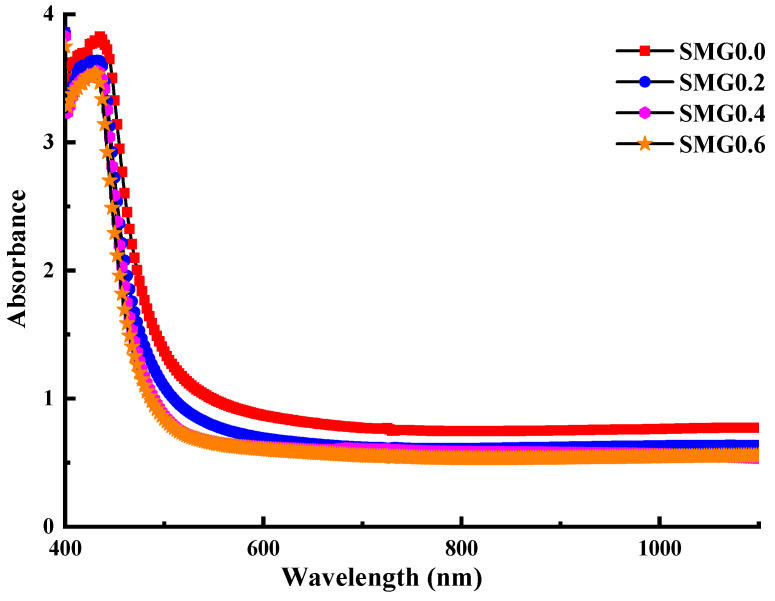
Spectra of Absorbance of glass samples.

**Figure 5:**
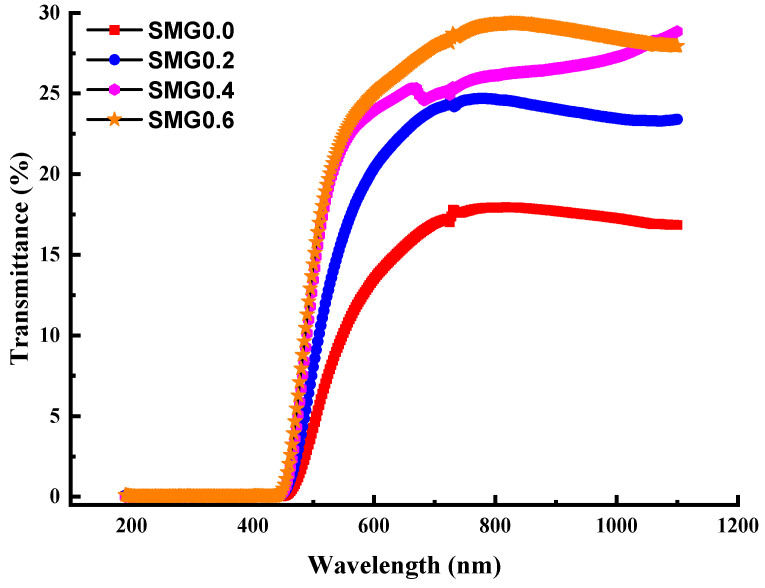
Spectra of transmittance of glass samples.

Figure 4 shows the absorbance of the samples in the(Tl_2_O_3_)_30_-(Li_2_O)_10_-(B_2_O_3_)_(60−*y*)_-(Sm_2_O_3_)*_y_* (*y* = 0, 0.2, 0.4, 0.6) glass system over 400–1100 nm. As seen, absorbance decreases with increasing Samarium (III) oxide contribution. The transmittance (T) spectra are shown in Figure 5 to vary depending on the wavelength values in the (Tl_2_O_3_)_30_-(Li_2_O)_10_-(B_2_O_3_)_(60−*y*)_-(Sm_2_O_3_)*_y_* (*y* = 0, 0.2, 0.4, 0.6) glass system with different doping rates. Additionally, in Figure 5 it is apparent that as Sm_2_O_3_ contribution increases the transmittance values also increase. This increase is observed more clearly at wavelengths longer than 600 nm. In the examined glass samples, an increase of 15% to 30% is observed in the transmittance values, with the Samarium (III) oxide weight content increasing from 0% to 0.6%. Rare earth oxides have a desired effect on the optical properties in the glassy systems, as glass transparency increases positively with the ratio of rare earth oxides in the glass.

The absorption coefficient α of the proposed glass system samples, depending on the absorbance values (A), is calculated with the help of the following equation [27]:

(1) $$\alpha =2.303\frac{A}{t}$$

**Figure 6:**
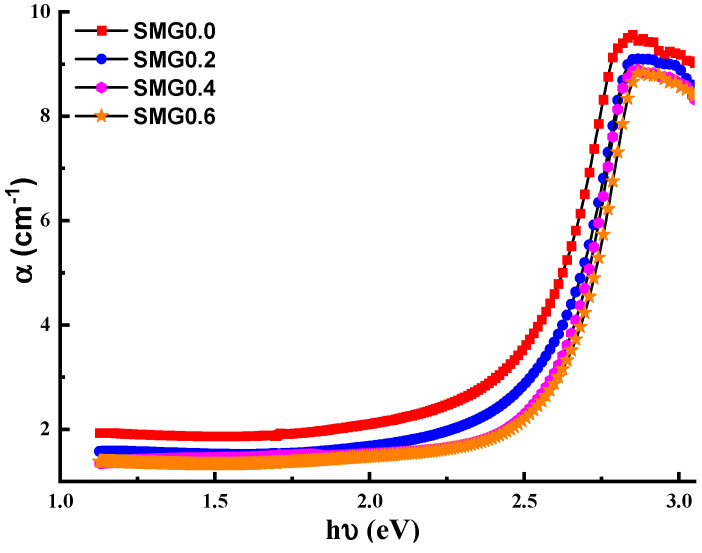
Absorption coefficient (α) of glass samples vs. incident energy.

In Equation (1), t shows the film thickness. The modification of the absorption coefficient (α) with incident photon energy (hv) for glass samples in the (Tl_2_O_3_)_30_-(Li_2_O)_10_-(B_2_O_3_)_(60−*y*)_-(Sm_2_O_3_)*_y_* system is depicted in Figure 6. As the content of Samarium (III) oxide in the solution increases, α value decreases, and the edges become extremely energized.

The extinction coefficient can be determined using (k) that is increasing exponentially [28]:

(2) $$k=\frac{\alpha \lambda }{4\pi }$$

**Figure 7:**
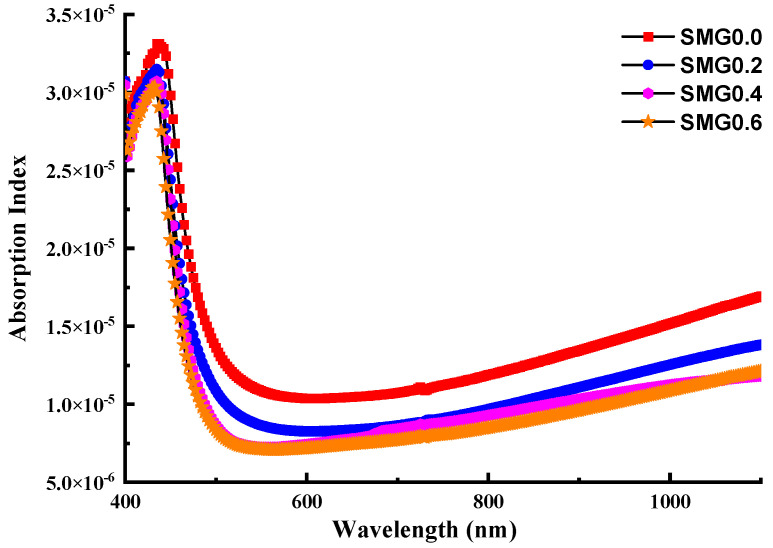
Absorption index of glass samples as a function of wavelength.

**Figure 8:**
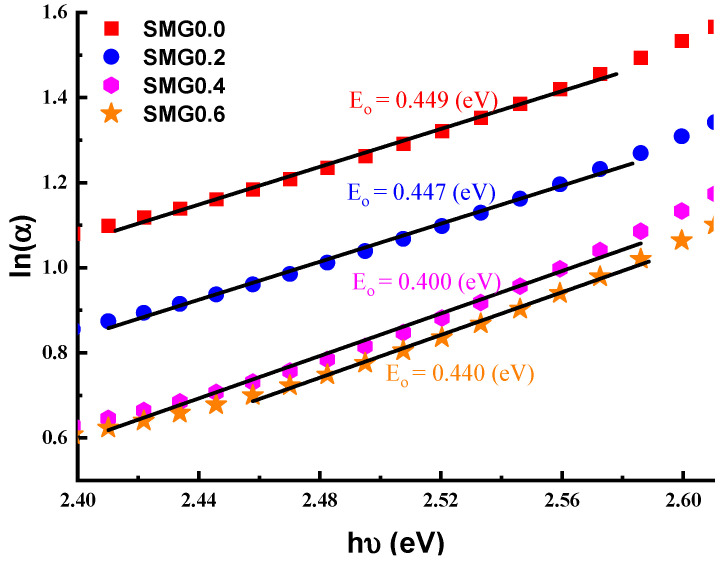
ln(α) of glass samples vs. the incident photon energy.

The dispersive absorption index for the (Tl_2_O_3_)_30_-(Li_2_O)_10_-(B_2_O_3_)_(60−*y*)_-(Sm_2_O_3_)*_y_* (*y* = 0, 0.2, 0.4, 0.6) glasses is shown in Figure 7. The action of k increases graphically with wavelength values, as shown in the diagram. At a particular temperature, the values of the absorption coefficient at the absorption edge follow the empirical Urbach law, which is given by the equation [21]:

(3) $$\alpha =\alpha_{o}e^{\frac{h\nu }{E_{o}}}\;$$

where *α_o_* and *E_o_* are the constant and the width of localized states, respectively. The plots of ln(α) vs. incident photon energy (hν) (Tl_2_O_3_)_30_-(Li_2_O)_10_-(B_2_O_3_)_(60−*y*)_-(Sm_2_O_3_)*_y_* (*y* = 0, 0.2, 0.4, 0.6) glass system are shown in Figure 8. As Samarium (III) oxide contribution increases in glass samples, there is a decrease in ln(α) values. Additionally, the graphic demonstrates that the electronic transition between states is accurate in the (Tl_2_O_3_)_30_-(Li_2_O)_10_-(B_2_O_3_)_(60−*y*)_-(Sm_2_O_3_)*_y_* glass samples.

### 3.3. Investigations on Nuclear Radiation Shielding Competencies

**Figure 9:**
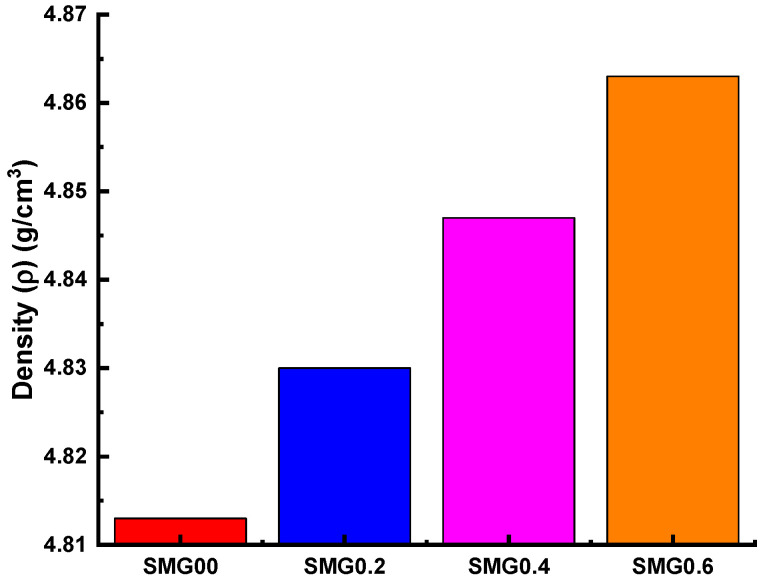
The density values of fabricated samples.

**Figure 10:**
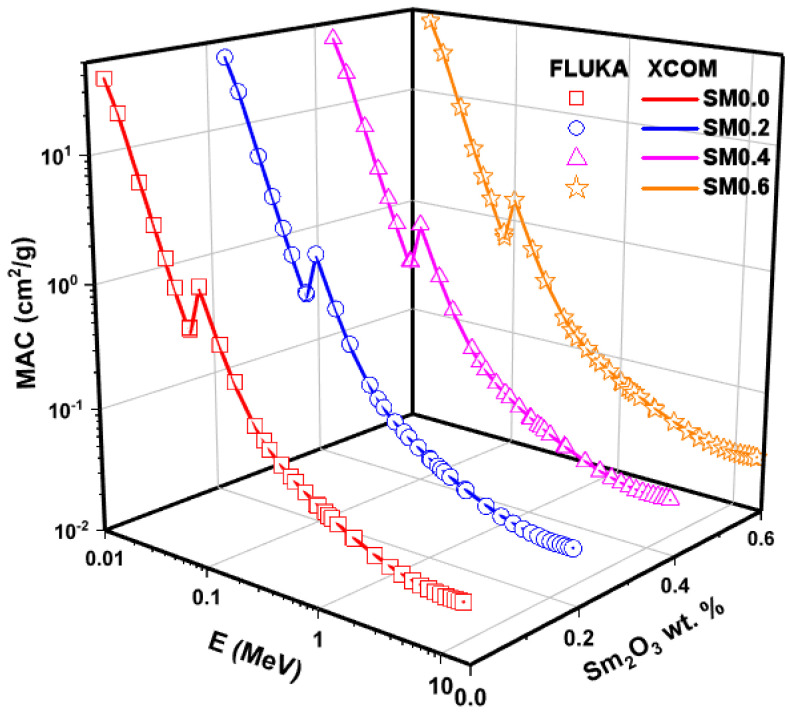
Mass attenuation coefficient by FLUKA and XCOM as a function of glass wt.% and photon energy.

**Figure 11:**
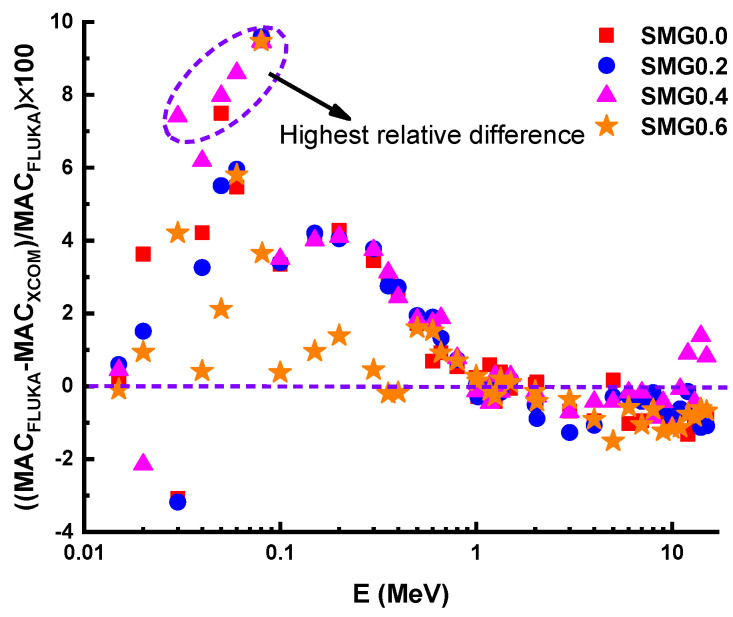
The relative difference of mass attenuation coefficient by XCOM and FLUKA simulation.

Figure 9 illustrates the density values (ρ) of the glass samples as a function of the Samarium (III) oxide addition. According to the measurements, as Samarium (III) oxide content increases, an increase is observed in density values. The density variation is explained by a rise in the amount of high density Sm in glass samples, while the amount of boron drops. The MAC values of the examined glass samples named SMG0.0, SMG0.2, SMG0.4, and SMG0.6 in the 0.015–15 MeV photon energy range, as calculated by the XCOM program, were compared with the FLUKA simulation code, as shown in Figure 10. As seen in Figure 10, the MAC values drop as photon energy levels increase. Additionally, a sharp increase in MAC values is found in the low energy zone (0.081 MeV), which is consistent with the effects of photoelectric supremacy in this range. In addition, cross-sectional values in this region are proportional to the Z^4^/E^3.5^ values [29]. Conversely, as we move towards the medium energies, a gradual decrease occurs due to the Compton effect. In addition, as Samarium (III) oxide contribution to glass samples is increased, the MAC values increase. The obtained results show that the XCOM and FLUKA results were consistent with each other, and this compliance is shown more clearly in Figure 11. As seen in Figure 11, the largest relative discrepancy between FLUKA (simulation) and XCOM findings is around 8%.

In order to determine the shielding properties of glass samples, HVL, TVL, and MFP values are crucial. By measuring the shielding potential of the different glass samples, glasses with the lowest HVL, TVL, and MFP values are shown to have the highest degree of shielding. The formulas are given by the following equations of *μ* coefficients [30]:

(4) $$\mathrm{HVL}=\frac{ln\left(2\right)}{\mu }\;\;\;$$

(5) $$\mathrm{TVL}=\frac{ln\left(10\right)}{\mu }\;\;\;\;\;\;\;$$

(6) $$\mathrm{MFP}=\frac{1}{\mu }\;\;\;\;\;$$

**Figure 12:**
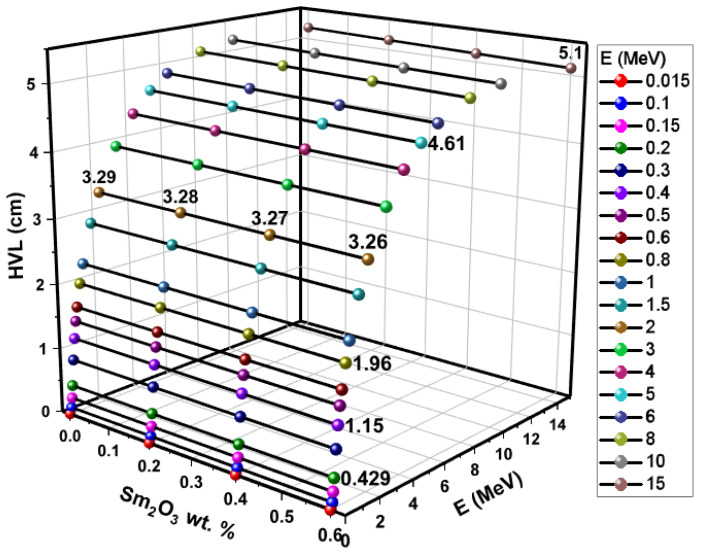
HVL results of glass samples as a function of glass composition and photon energy.

The HVL values of the glass samples SMG0.0, SMG0.2, SMG0.4, and SMG0.6 are shown in Figure 12 according to the photon energy values and considered Samarium (III) oxide contributions. These values are also shown in Table 2. It is clear that the change in HVL values is significantly dependent on the Samarium (III) oxide content. HVL values decrease as Samarium (III) oxide content increases, as expected. According to the results obtained, the glass sample SMG0.6 has the lowest HVL value. Conversely, as the energy values of gamma photons increase, an increase is observed in HVL values. Clearly, the SMG0.6 glass sample is the most effective of the glass samples considered in this work in reducing gamma radiation.

**Figure 13:**
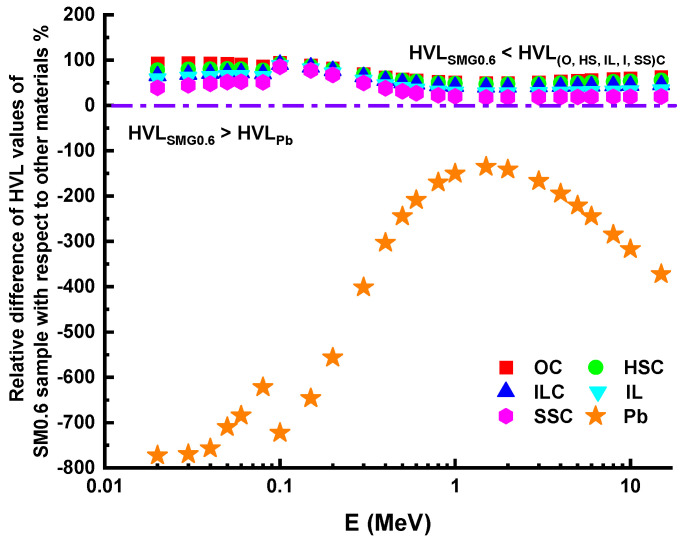
HVL relative differences of SM0.6 sample and HVL relative differences of other materials.

The HVL values of the SM0.6 glass sample, which has the best shielding performance, are compared to those of many other materials, as shown in Figure 13. These materials are widely used in nuclear applications as protective materials. The brief order of the materials mentioned above can be listed as O-C (ordinary-concrete), lead (Pb), H-S-C (hematite-serpentine-concrete), I-C (ilmenite-concrete), I-L-C (ilmenite-limonite-concrete), and S-S-C (steel-scrap-concrete) [31]. As seen in Figure 13, the HVL values of the SM0.6 glass sample are lower than those of HS-C, O-C, I-C, I-L-C, and S-S-C. Compared to lead, it has better shielding properties.

**Figure 14:**
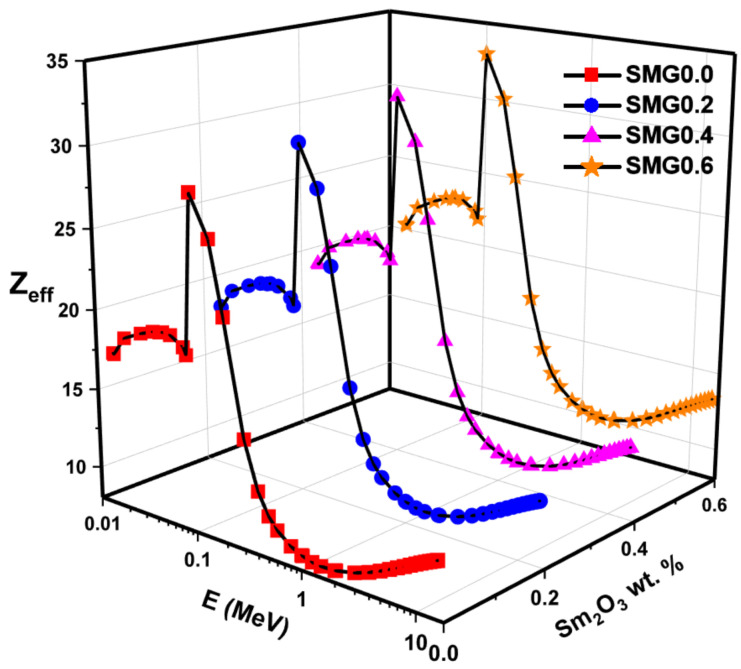
Z_eff_ of glass samples as a function of the photon energy.

**Figure 15:**
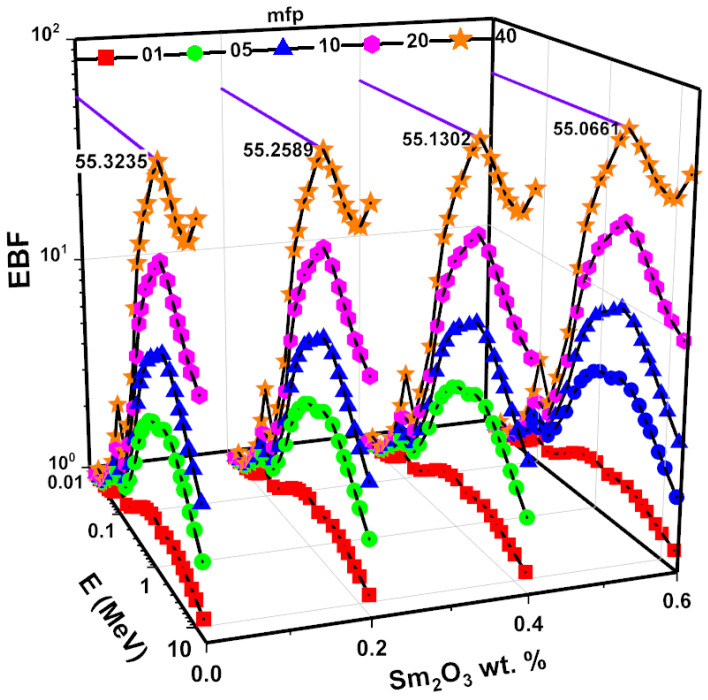
EBF for samples as a function of energy at 1, 5, 10, 20, and 40 mfp.

**Figure 16:**
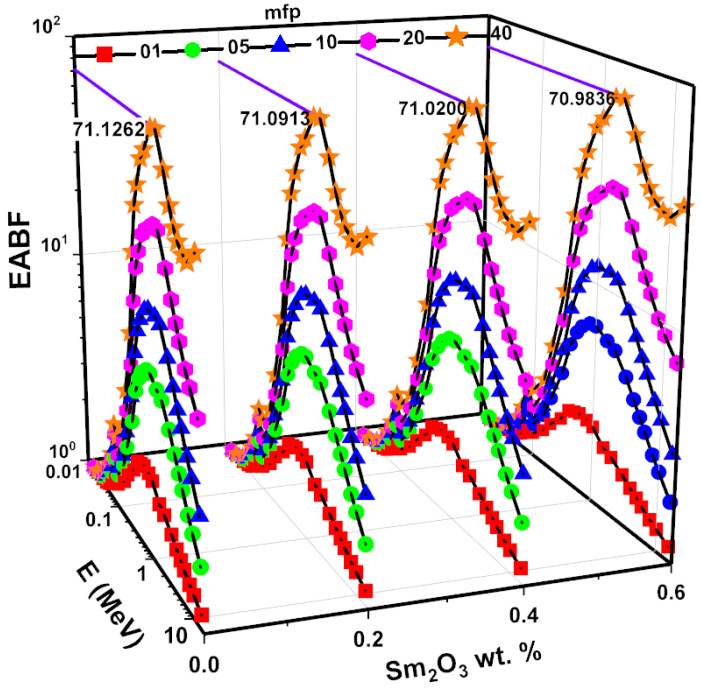
EABF for samples as a function of energy at 1, 5, 10, 20, and 40 mfp.

The Z_eff_ values, dependent on the photon energy of the examined glass samples, are given in Figure 14. Z_eff_ values indicate the radiation absorption capacity of a glass sample. An increase in Z_eff_ values is evident from SMG0.0 to SMG0.6. In addition, Z_eff_ values behave differently depending on the processes of interactivity between photons and matter. The Z_eff_ values for glass samples are greater than threshold in the low-energy zone, and glass sample peaks occur around the Tl K-edge. The Z_eff_ energy dependence decreases when the crossover approaches the medium and high energy ranges. The Z_eff_ values for the SMG0.6 glass sample differ in the range of 10 to 33. Appropriate assays were built to collect data to assess the buildup variables. The differences found in the examined glass samples are seen in Figure 15 and Figure 16, based on energy values and different penetration depth (1, 5, 10, 20, and 40 mfp). As shown in Figure 15 and Figure 16, three particle-photon interactions affected the EBF and EABF counts. For the SMG0.0, SMG0.2, SMG0.4, and SMG0.6 samples, in the low energy region of 0.015 MeV to 0.1 MeV, the EBF and EABF energies are highest since Tl K-absorption spectrum lies in this region. Although EAF values are steady between 0.2 MeV and 7 MeV energy values, they rise more rapidly after 6 MeV (particularly at high penetration depths). It is demonstrated that at intermediate energies where Compton scattering occurs, the EBF and EABF glass sample values change proportionally to Z_eq_ (Figure 15 and Figure 16.) Photon buildup is small for an SMG0.6 glass sample with maximum Z_eq_ values. Between 7 MeV and 15 MeV, the EBF and EABF values of the examined samples increased more rapidly, owing to the dominance of pair production and its dependence on Z^2^ [32]. Figure 15 and Figure 16 display substantially higher EBF and EABF values as penetration depth (1–40 MFP) increases. This suggests an improved sample thickness in secondary photons. The SM0.6 sample has the minimum EBF and EABF values at moderate energy, which is critical for radiation applications, while the SMG0.0 sample exhibited the highest. This demonstrates that the sample SMG0.6 is more successful at absorbing gamma radiation than other samples.

**Figure 17:**
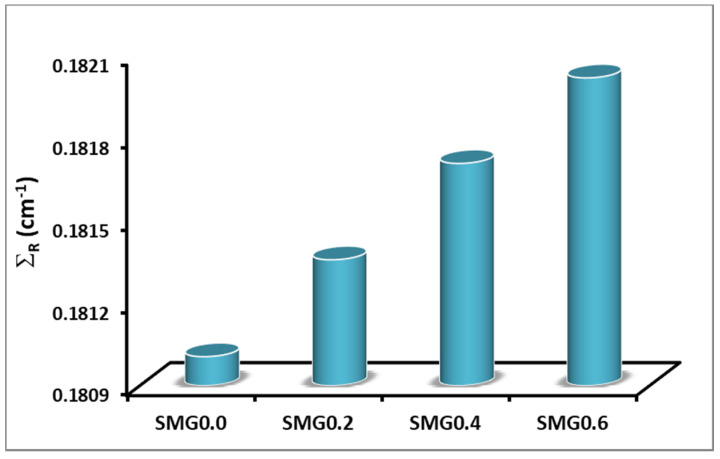
Effective removal cross section of samples for fast neutrons.

The effective removal cross section (ΣR) values of each glass sample were determined for fast neutrons. The glass samples were classified according to their MAC values. It is recognized that glass samples with high ΣR values provide greater neutron radiation safety. The energy lost by charged particles slows as they move through the substance, and this result is owed to the kinetic energy shifts of the particles; with lowered energy losses, the rate of ionization increases. It is important to determine whether the studied materials can withstand the impact of high-energy charged particles [17,33]. The measured effective removal cross-section values (Σ_R_) for all glass samples analyzed are shown in Figure 17. As the number of protons in the material increases, the neutron shielding ability of the material decreases. According to Figure 17, as expected, 0.6% of glasses containing the highest concentration of Samarium (III) oxide has the highest defense against neutrons.

## 4. Conclusions

The effect of Samarium (III) oxide content on the optical properties of the glass sample (Tl_2_O_3_)_30_-(Li_2_O)_10_-(B_2_O_3_)_(60−*y*)_-(Sm_2_O_3_)*_y_* (*y* = 0, 0.2, 0.4, 0.6) was shown. The SMG0.0 glass system was found to have three peaks. For *x* = 0.2, 0.4, 0.6 at 2355 cm^−1^, referred to in Sm-O bonds, a new fourth measurable peak emerged. For the glass device with increasing Samarium (III) oxide, an improvement of the optical transmission was observed. At incident wavelengths longer than 600 nm, this rise was clearly seen. In addition, MAC values in the 0.015-15 MeV photon energy range were obtained using the XCOM program with FLUKA code. Using MAC values, the HVL, Zeff, Z_eq_, EBF, and EABF values were estimated. As the results demonstrate, the small increase in Samarium (III) oxide content in the glass samples also caused a small increase in the MAC and Z_eff_ values. However, as Samarium (III) oxide contribution increased, HVL, EBF, and EABF values decreased, and MAC, Zeff, and Z_eq_ values increased. These findings indicated that adding Samarium (III) oxide to glass samples improved their gamma shielding characteristics. As a result, it may be proposed that SM0.6 (which includes the greatest concentration of Samarium (III) oxide) has a high capacity for radiation shielding. The research hypothesized that increasing the Samarium (III) oxide contribution would greatly improve the produced glass samples’ radiation attenuation and optical properties. As a result, our findings confirm the hypothesis in terms of progress toward enhancing the nuclear radiation shielding characteristics of synthesized glasses in a manner consistent with other crucial parameters such as optical and structural qualities.

## Figures and Tables

**Table 1:** IR band of (Tl_2_O_3_)_30_-(Li_2_O)_10_-(B_2_O_3_)_(60-y)_-(Sm_2_O_3_)_y_ (*y =* 0, 0.2, 0.4, 0.6) glass samples.

Code	SMG0.0	SMG0.2	SMG0.4	SMG0.6	IR Band Assignments	Reference
IR band positions	1035	1049	929	1049	(B–O stretching of tetrahedral ${\mathrm{BO}}_{4}^{-}$ units)	[24]
	1643	1643	1643	1643	Crystal water with H−O−H bending mode	[25]
	3434	3434	3434	3434	B–OH	[25]
	-	2355	2342	2355	Sm–O	[26]

**Table 2:** The results of the mean free path-MFP and Tenth value layer -TVL of the studied samples.

E (MeV)	TVL				MFP			
	SMG0.0	SMG0.2	SMG0.4	SMG0.6	SMG0.0	SMG0.2	SMG0.4	SMG0.6
0.015	0.01189	0.01181	0.01172	0.01165	0.00516	0.00513	0.00509	0.00506
0.02	0.02148	0.02135	0.02121	0.02108	0.00933	0.00927	0.00921	0.00916
0.03	0.06211	0.06172	0.06133	0.06096	0.02697	0.02680	0.02663	0.02648
0.04	0.13194	0.13111	0.13033	0.12955	0.05730	0.05694	0.05660	0.05626
0.05	0.23451	0.23041	0.22632	0.22251	0.10185	0.10007	0.09829	0.09663
0.06	0.37029	0.36391	0.35745	0.35151	0.16081	0.15805	0.15524	0.15266
0.08	0.72806	0.71634	0.70504	0.69406	0.31619	0.31110	0.30619	0.30143
0.1	0.31915	0.31718	0.31502	0.31316	0.13861	0.13775	0.13681	0.13600
0.15	0.80731	0.80257	0.79774	0.79325	0.35061	0.34855	0.34645	0.34450
0.2	1.44666	1.43895	1.43132	1.42403	0.62828	0.62493	0.62161	0.61845
0.3	2.76378	2.75246	2.73964	2.72748	1.20029	1.19538	1.18981	1.18453
0.4	3.85814	3.84146	3.82491	3.80926	1.67557	1.66833	1.66114	1.65434
0.5	4.72270	4.70144	4.68495	4.66953	2.05104	2.04181	2.03465	2.02795
0.6	5.43153	5.41119	5.39160	5.37264	2.35889	2.35005	2.34154	2.33331
0.8	6.58785	6.56466	6.54164	6.52011	2.86107	2.85100	2.84100	2.83165
1	7.55066	7.52408	7.49769	7.47420	3.27921	3.26767	3.25621	3.24600
1.5	9.48661	9.45509	9.42380	9.39279	4.11998	4.10629	4.09270	4.07924
2	10.93258	10.89410	10.85589	10.82017	4.74796	4.73125	4.71465	4.69914
3	13.04279	12.99334	12.94777	12.90165	5.66441	5.64294	5.62314	5.60312
4	14.48409	14.42874	14.37379	14.31783	6.29036	6.26632	6.24246	6.21815
5	15.48752	15.42302	15.35899	15.29857	6.72615	6.69813	6.67033	6.64408
6	16.18436	16.11104	16.04369	15.97472	7.02878	6.99694	6.96769	6.93773
8	16.98294	16.89917	16.82201	16.74295	7.37560	7.33922	7.30571	7.27137
10	17.29608	17.21031	17.12522	17.04430	7.51159	7.47434	7.43739	7.40225
15	17.23377	17.13608	17.04534	16.95276	7.48453	7.44210	7.40270	7.36249

## Data Availability

The data presented in this study are available on request from the corresponding author.
